# Supplementary material for: A hemoprotein with a zinc-mirror heme site ties heme availability to carbon metabolism in cyanobacteria
Source: Nat Commun. 2024 Apr 12;15:3167. doi: 10.1038/s41467-024-47486-z (PMC11014987; doi:10.1038/s41467-024-47486-z)
Supplement: Supplementary file 1 — Supplementary Information [file 41467_2024_47486_MOESM1_ESM.pdf]

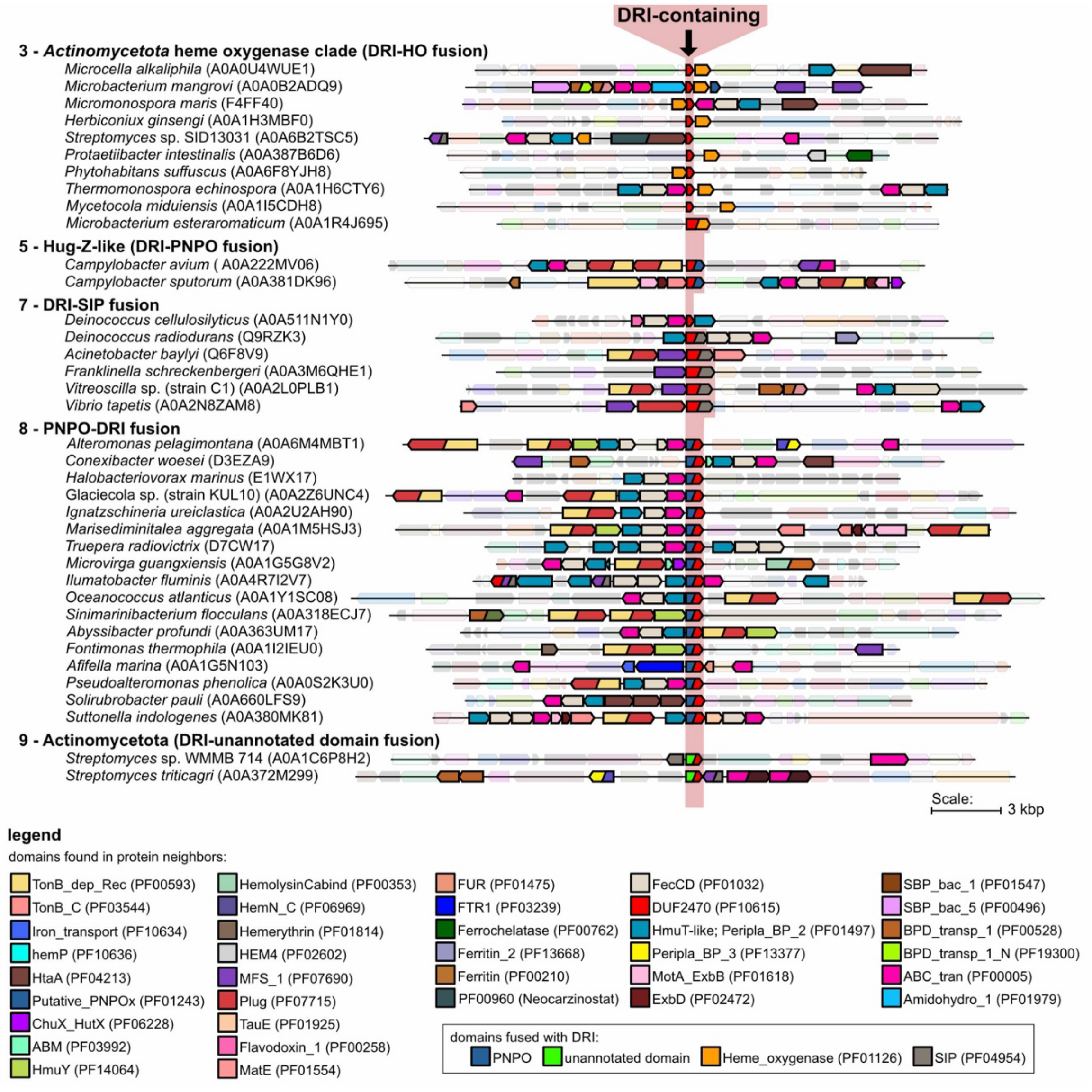

Supplementary Fig. 1: Gene-neighborhood analysis of DRI-containing genes. Each number on the left corresponds to a numbered clade in Fig. 1a. Each species name is followed by the UniProt protein ID for the corresponding DRI-containing protein. Genes are colored based on the domains identified in encoded proteins according to the legend.

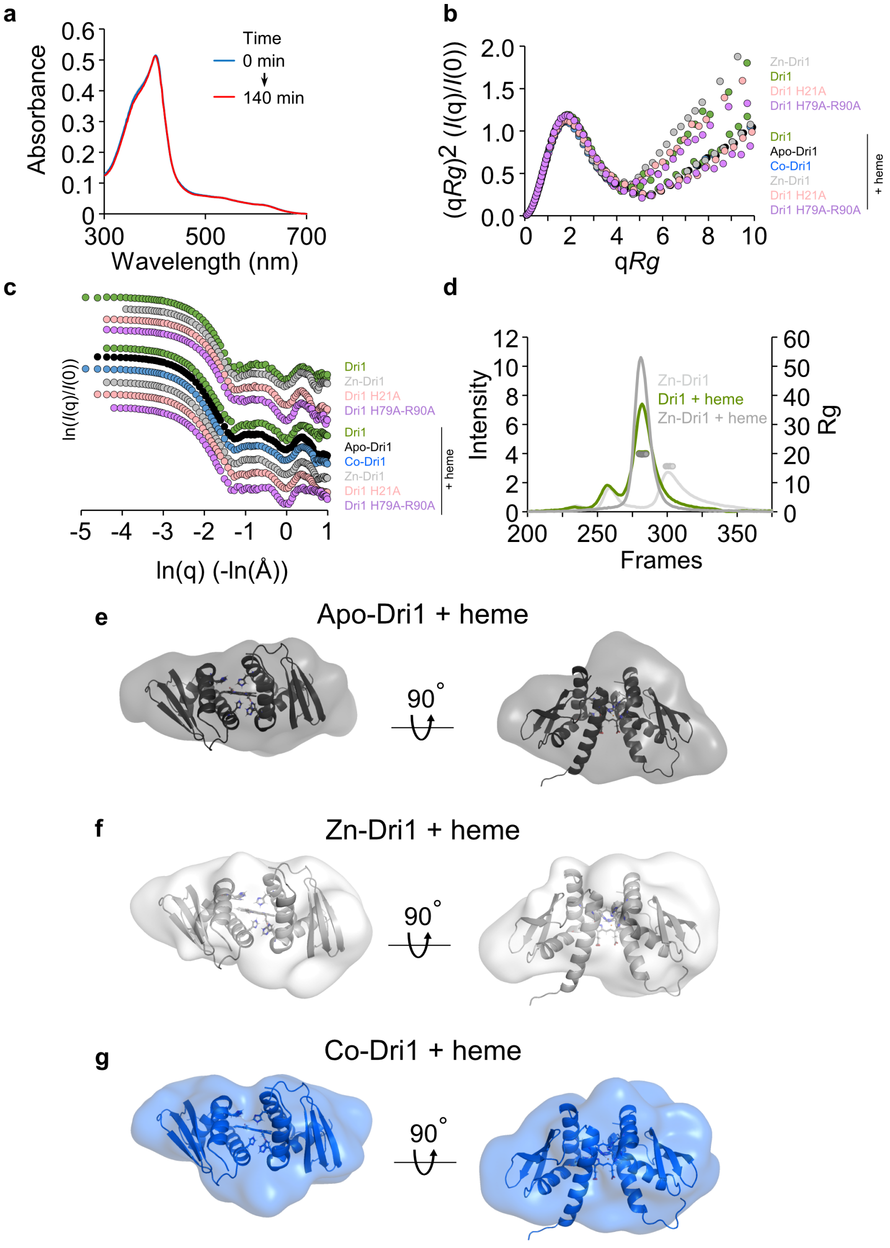

Supplementary Fig. 2: SAXS characterization of Dri1 and variants. a, UV- Vis absorption spectra kinetics of heme-bound Dri1 in the presence of ascorbate as electron donor. Kinetics was started after addition of ascorbate and monitored every 5 min for 140 min. b, Dimensionless Kratky and c, log-log plots of SAXS data from Fig. 3. Heme binding and subsequent homodimerization results in similar changes of flexibility in all variants. d, SEC-SAXS chromatograms using a Biozen dSEC-2 column. R_g of the frames used for subsequent processing are displayed as data points. e-g, DENSS envelopes calculated from SEC-SAXS samples of apo-Dri1 (e), Zn-Dri1 (f), and Co-Dri1 (g). DENSS envelopes were calculated from SAXS data to $qmax$ = 1.0 Å^{-1}. Source data are provided as a Source Data file.

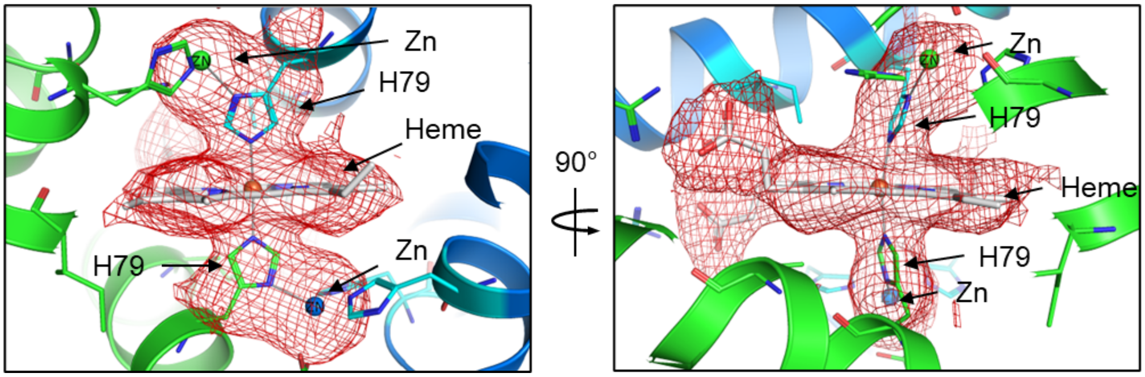

Supplementary Fig. 3: Difference Fourier map of the heme site (shown in red mesh) of WT Dri1 crystal structure before including heme, zinc ions and His79 side chains from each monomer (the two distinct monomers are depicted in green and blue, respectively). Heme, zinc ions and His79 side chains were subsequently overlaid onto the difference Fourier map. Two different orientations (90° rotation) are displayed.

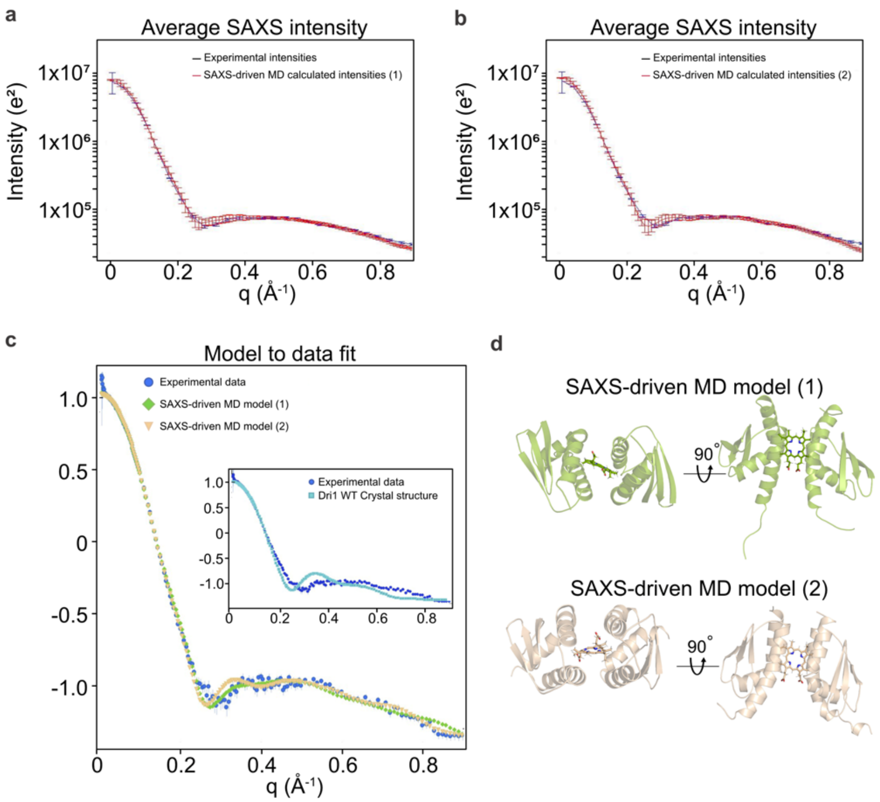

Supplementary Fig. 4: SAXS-driven and free-MD simulations of Dri1 a-b, Average intensities calculated from SAXS-driven MD trajectories (5 ns to 30 ns). Dri1 SAXS-driven MD average intensity plots with starting structure as (a) Dri 1 crystal structure (b) Free-MD 100 ns model. c-d, Best fitting models from the SAXS-driven MD trajectories based on $\chi 2$ values generated from FoXS server compared to the Dri1 crystal structure fit against the SAXS data (inset). Source data are provided as a Source Data file.

a

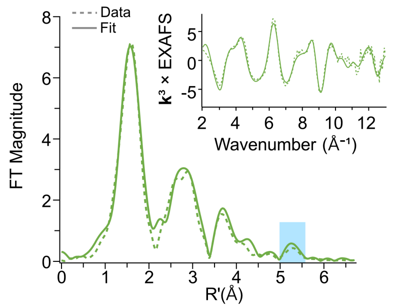

b

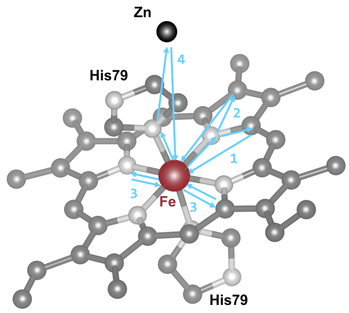

c

Fe EXAFS

WT

Path	Coordination	R(Å)[a]	σ^2 (Å^2)[b]	ΔE	K range	R-factor	χ^2_{red}
N	6	2.044(7)	631	1.9	2-13	0.028	36.8
C	2	2.94(4)	232				
C	8	3.07(1)	232				
N-C	26	3.26(2)	1220				
C	4	3.77(3)	347				
C	8	4.1(1)	2189				
N-C	16	4.32(2)	706				
N-C	24	5.3(1)	1038				
Zn	1	5.53(6)	340				
N-C	26	5.69(6)	784				

R range	N_{var}	N_{idp}
1-6	20	34.6

[a]The Fe-Zn scattering interaction was modeled with a single Fe-Zn path.

d

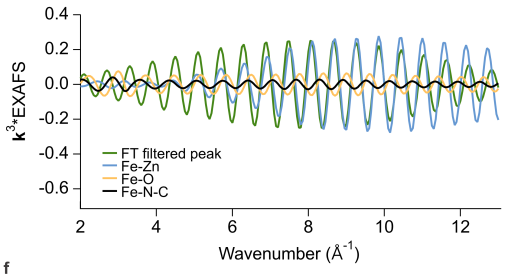

e

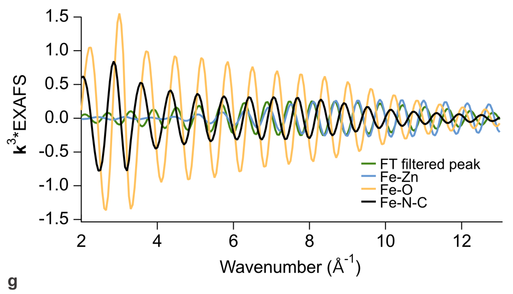

f

Fe EXAFS

Apo

Path	Coordination	R(Å)[a]	σ^2 (Å^2)[b]	ΔE	K range	R-factor	χ^2_{red}
N	5	2.020(4)	390	-0.8	2-12	0.011	8.8
N	1	2.51(3)	607				
C	2	2.95(6)	255				
C	8	3.06(1)	255				
N-C	24	3.27(1)	381				
C	4	3.75(2)	339				
C	12	4.30(2)	43				
N-C	16	4.38(2)	107				
N-C	16	5.4(1)	1059				

R range	N_{var}	N_{idp}
1-5.6	18	29

g

Fe EXAFS

WT

Path	Coordination	R(Å)[a]	σ^2 (Å^2)[b]	ΔE	K range	R-factor	χ^2_{red}
N	2	2.12	207	2.5	2-13	0.03	16.6
N	4	2.01	338				
C	2	2.94	228				
C	8	3.07	228				
N-C	26	3.26	1299				
C	4	3.77	343				
C	8	4.07	1745				
N-C	16	4.32	741				
N-C	24	5.33	1207				
N-C	26	5.7	793				

R range	N_{var}	N_{idp}
1-6	0	34.6

h

Fe EXAFS

WT

Path	Coordination	R(Å)[a]	σ^2 (Å^2)[b]	ΔE	K range	R-factor	χ^2_{red}
N	6	2.04	631	1.9	2-13	0.033	17.9
C	2	2.94	232				
C	8	3.07	232				
N-C	26	3.26	1220				
C	4	3.77	347				
C	8	4.09	2189				
N-C	16	4.32	706				
N-C	24	5.33	1038				
N-C	26	5.69	784				

R range	N_{var}	N_{idp}
1-6	0	34.6

Supplementary Fig. 5: A comparison of the Fourier-back transform of the peak corresponding to the Fe-Zn backscattering interaction with other possible scattering interactions. a, The Fourier-Transform plot of the WT Fe EXAFS (dashed line) and 6 coordinate first shell fit (solid line), the blue box highlights the peak subject to Fourier-filtering. (Inset) $\mathbf{k}^3$-weighted EXAFS. b, A simplified structural schematic of the local structure around the Fe atom when Zn is bound. Shown in cyan are examples of the types of multiple scattering interactions which contribute to each of the four multiple scattering paths employed in the EXAFS fitting. C = grey, N = light grey, Fe = red, Zn = black. Representative angles obtained from the crystal structure used in the EXAFS fitting: 1) 129°, 2) 165°, 3) 161°, 4) 145°. c, Fe EXAFS least-squares fitting parameters for Dri1 with a single six-coordinate N path in the first shell. The Fe-Zn scattering interaction was modelled with a single Fe-Zn path. d, A comparison of the Fourier-filtered peak highlighted in a, with scattering interactions produced by a single Fe-Zn, Fe-O or Fe-N-C path at a distance of 5.6 Å. All EXAFS variables from the best fit were used to simulate the paths ($S_0^2 = 1$, $\Delta E = 2.5$, $\sigma^2 = 0.00365$, $\Delta R = 0$). e, A comparison of the Fourier-filtered peak highlighted in a, with scattering interactions produced by a single Fe-Zn path or 25 Fe-O or Fe-N-C paths. To compensate for the higher coordination numbers of the Fe-O and Fe-N-C paths, the σ^2 was raised to 0.01 for those two paths only. f, Fe EXAFS least-squares fitting parameters for apo-Dri1 from Fig. 3f. g, Fe EXAFS least-squares fitting parameters for Dri1 with Fe-N paths separated into two components in the first shell (4 equatorial Fe-N paths from the heme ring and 2 Fe-N paths from the two His79), and by excluding the Zn path. h, same as g but with a single six-coordinate N path in the first shell. [a]The estimated standard deviations for distance are on the order of ± 0.02 Å. [b]Values of σ^2 have been multiplied by 10^5. The value of S_0^2 was set to 1 for all fits. Coordination numbers have an error of ± 20%. Source data are provided as a Source Data file.

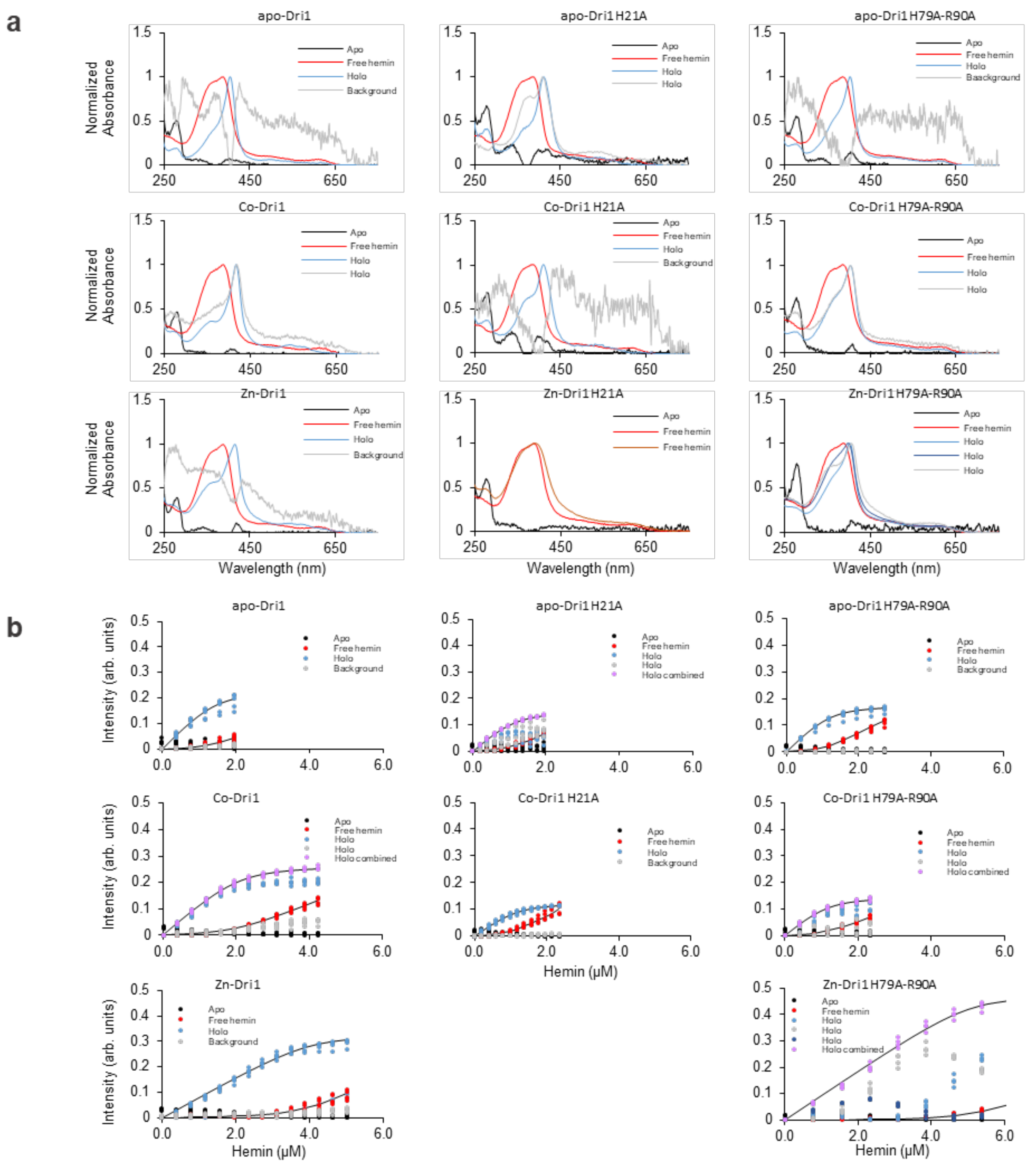

c

Variant	*p* (µM)	K_d (µM)	*a*	*b*	# of hemin additions	Final hemin conc (µM)	Final protein conc (µM)
apo-Dri1	1.50 ± 0.10	0.15 ± 0.04	6.3 ± 0.8	17 ± 2	5	1.95	4.95
Co-Dri1	2.14 ± 0.12	0.17 ± 0.03	7.8 ± 0.3	17 ± 2	11	4.26	4.88
Zn-Dri1	4.2 ± 0.4	0.11 ± 0.02	12.2 ± 1.1	13.4 ± 1.1	13	5.00	4.86
apo-Dri1 H21A	1.17 ± 0.09	0.16 ± 0.06	7.3 ± 0.6	15.0 ± 1.1	10	1.95	4.95
Co-Dri1 H21A	1.2 ± 0.2	0.16 ± 0.06	9.3 ± 1.4	12.8 ± 1.5	12	2.34	4.94
apo-Dri1 H79A-R90A	1.21 ± 0.06	0.15 ± 0.03	6.68 ± 0.13	13.86 ± 0.16	7	2.72	4.93
Co-Dri1 H79A-R90A	1.20 ± 0.12	0.15 ± 0.02	7.8 ± 0.9	18 ± 2	6	2.34	4.94
Zn-Dri1 H79A-R90A	5.19 ± 0.13	0.09 ± 0.02	10.6 ± 0.6	22.5 ± 1.4	8	6.13	4.90

Supplementary Fig. 6: Heme binding analysis of Dri1 and mutants. a, Spectral components from MCR-ALS deconvolutions. Apoprotein (black), free hemin (red), holoprotein (blue), and background (grey/navy) components were determined. For Co-Dri1, apo-Dri1 H21A, and Zn- or Co- Dri1 H79A-R90A data sets, background spectra resembled that of the holoprotein, and so, the corresponding holoprotein concentration profiles in panel b were summed together. For Zn-Dri1 H21A, no holoprotein component was identified. b, Non-linear least squares fits of components decomposed from MCR-ALS analyses. Average fits of all data sets (n = 4 - 6) are displayed in black lines. c, Parameters calculated from the hemin titration of apoprotein. p_0 = estimated concentration of "active" protein (µM), K_d = binding constant to hemin. α and β = arbitrary scaling factors to convert the concentrations into molar concentrations. Source data are provided as a Source Data file.

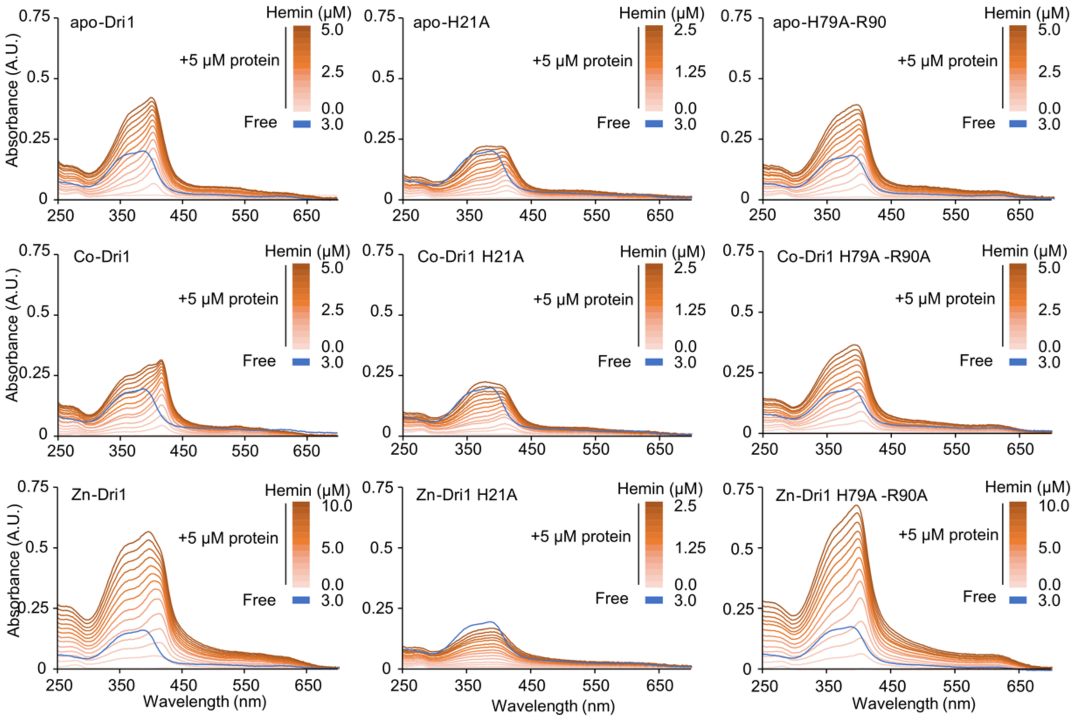

Supplementary Fig. 7: Absorption spectra of Dri1 and variants with hemin. Hemin titration of apo-Dri1 (WT and variants, 5 µM) or with addition of Co^{2+} (Co-Dri1) or Zn^{2+} (Zn-Dri1). UV-Vis spectra were acquired three minutes after each addition of hemin. Spectra in blue are of free hemin in buffer (~3 µM). Source data are provided as a Source Data file.

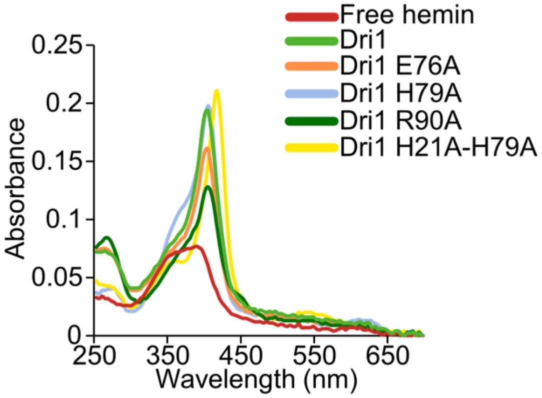

Supplementary Fig. 8: UV-Vis absorption spectra of Dri1 and amino acid variants with hemin. Source data are provided as a Source Data file.

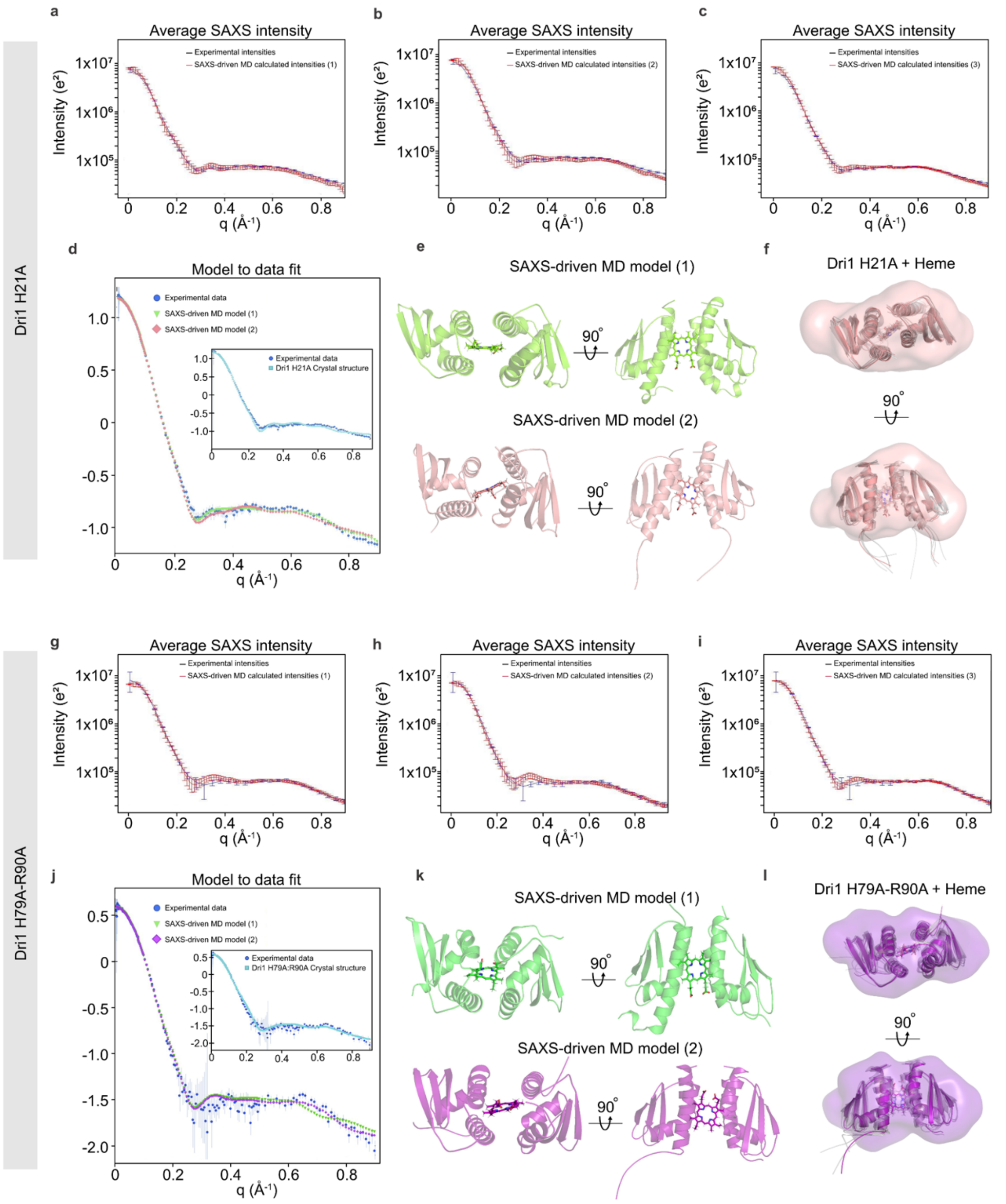

Supplementary Fig. 9: SAXS-driven and free-MD simulations of Dri1 variants. a-c H21A SAXS-driven MD average intensity plots with starting structure as (a) Dri1 crystal structure with H21A mutation, (b) Free-MD 100 ns model from Dri1 crystal structure with H21A mutation, and (c) Dri1 H21A crystal structure. d, SAXS-driven MD trajectories based on $\chi2$ values generated from FoXS server compared to the Dri1 H21A crystal structure fit against the SAXS data (inset). e, SAXS-driven MD model 1 from (b) and model 2 from (c). f, DENSS envelope calculated from SEC-SAXS samples of Dri1 H21A superimposed with SAXS-driven MD models from Dri1 H21A crystal structure. DENSS envelopes were calculated from SAXS data to $qmax = 1.0$ Å^{-1}. g-i, Dri1 H79A-R90A SAXS-driven MD average intensity plots with starting structure as (g) Dri1 crystal structure with H79A-R90A mutation, (h) Free-MD 100 ns model from Dri1 crystal structure with H79A-R90A mutation, and (i) Dri1 H79A-R90A crystal structure. j, SAXS-driven MD trajectories based on $\chi2$ values generated from FoXS server compared to the Dri1 H79A-R90A crystal structure fit against the SAXS data (inset). k, SAXS-driven MD model 1 from (h) and model 2 from (i). l, DENSS envelope calculated from SEC-SAXS samples of Dri1 H79A-R90A superimposed with SAXS-driven MD models from Dri1 H79A-R90A crystal structure. DENSS envelopes were calculated from SAXS data to $qmax = 1.0$ Å^{-1}. Source data are provided as a Source Data file.

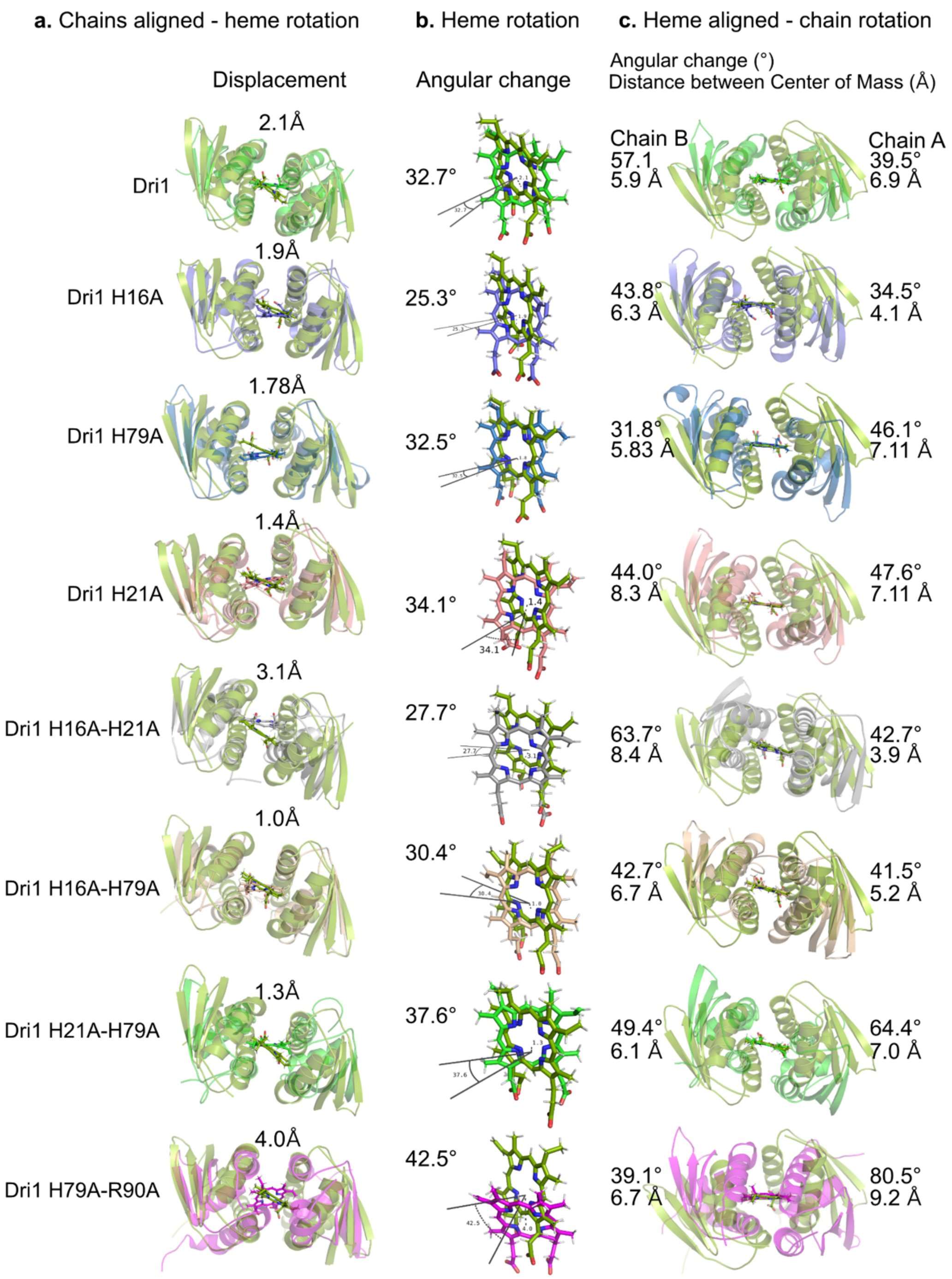

Supplementary Fig. 10: Last free-molecular dynamic (MD) frames (100 ns). a, Alignments of the last free- (MD) frames (100 ns) of WT and variants with the Dri1 crystal structure. Displacement of heme between the two chains is indicated for each protein. b, Heme rotation measurements from (a). c, Analysis of chain rotation around heme by alignment of the hemes. The angular change and the displacement between Chain A of Dri1 crystal structure v/s Chain A of the last frame of free-MD simulation and Chain B of Dri1 crystal structure v/s Chain B A of the last frame of free-MD simulation were calculated by taking center of mass of both chains as the reference point.

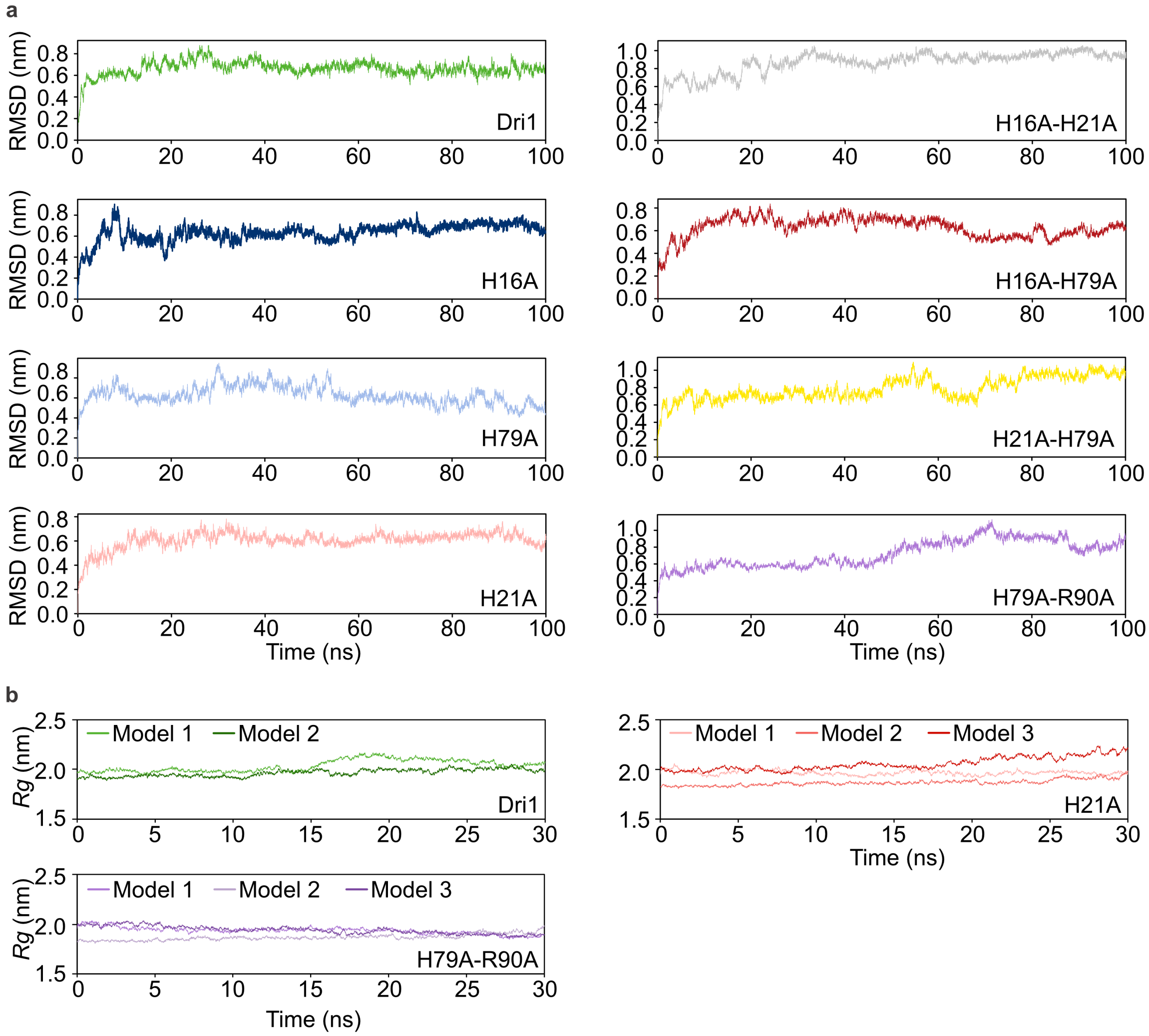

Supplementary Fig. 11: Stability assessment of MD simulations. a, RMSD plots of free-MD simulations for Dri1 and mutants showing stable trajectory over the course of a simulation of 100 ns. b, Gyration radius (Rg) plots of SAXS-driven MD showing stable radius over the course of a simulation of 30 ns for Dri1, Dri1 H21A and Dri1 H79A-R90A using different starting structure models. Source data are provided as a Source Data file.

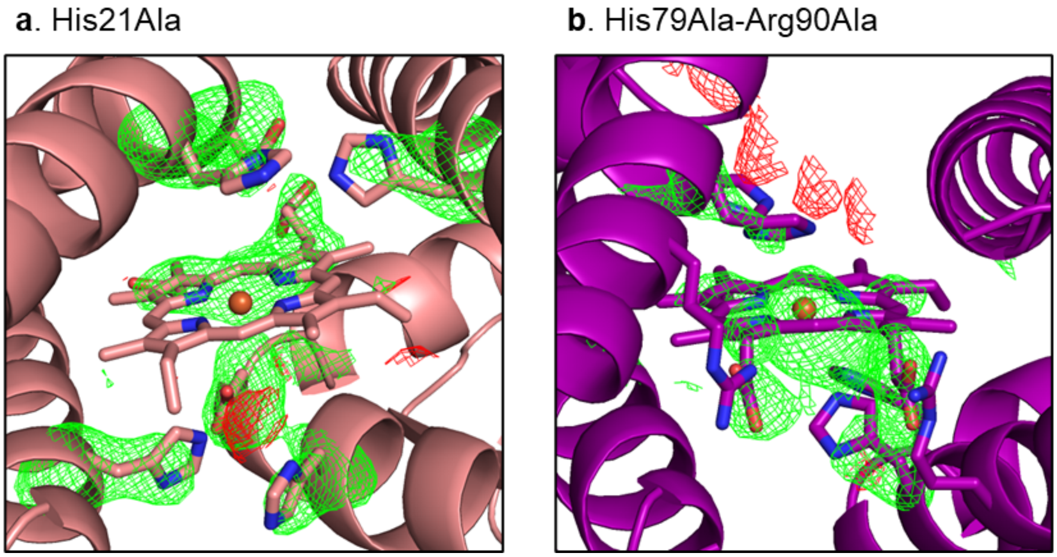

Supplementary Fig. 12: Polder OMIT maps of Dri1 variants. a, Polder OMIT map of His21Ala Dri1-Heme contoured at 2.8σ. Heme, Asp20, His residues, and bulk solvent in the region were excluded. Positive and negative $mF_{obs}-DF_{calc}$ difference densities up to 2 Å from heme, Asp20, and His residues are displayed in green and red, respectively. b, Polder OMIT map of His79Ala-Arg90Ala Dri1-Heme contoured at 2.2σ. Heme, His residues, and bulk solvent in the region were excluded. Positive and negative $mF_{obs}-DF_{calc}$ difference densities up to 2.5 Å from heme and His residues are displayed in green and red, respectively.

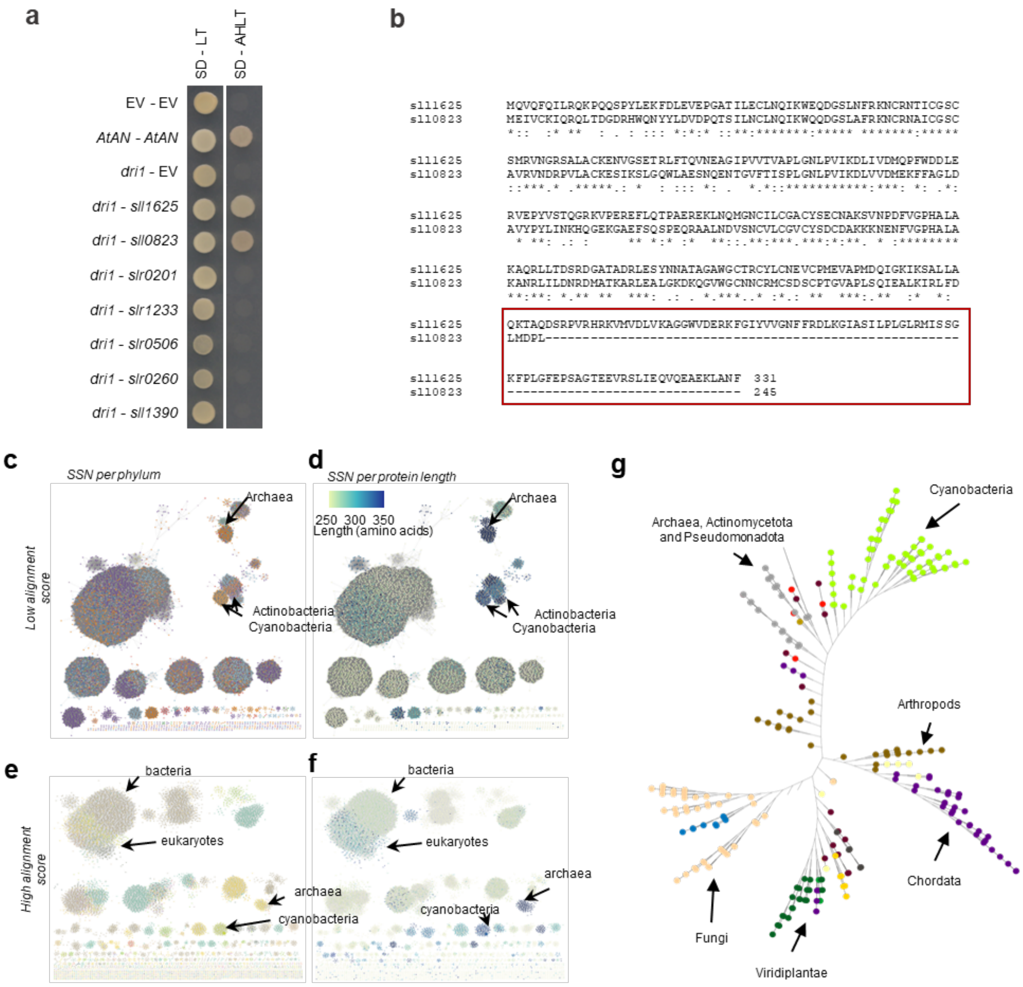

Supplementary Fig. 13: Dri1 interaction analysis with SDH subunit by Y2H and Succinate Dehydrogenase subunit B analysis (Iron-sulfur cluster subunit). a, Dri1 was fused with the Gal4 DNA-binding domain (BD). Putative interactors were fused with the Gal4 activation domain (AD). SD-LW indicates SD without Leu and Trp. SD-LWHA indicates SD without Leu, Trp, His, and Ade. Self-interaction of AtAN was used as a positive control. EV, empty vector. b, amino acid sequence alignment between SdhB1 (sll1625) and SdhB2 (sll0823) showing C-terminal extension (red box) in SdhB1 (sll1625) containing a single histidine residue (blue circle). c, SSN of SdhB family (IPR004489) colored by phylum with alignment score of 70. d, SSN of SdhB family (IPR004489) colored by protein length with alignment score of 70. e, SSN of SdhB family (IPR004489) colored by phylum with alignment score of 100. f, SSN of SdhB family (IPR004489) colored by protein length with alignment score of 100. g, phylogenetic tree of SdhB proteins (IPR004489). Source data are provided as a Source Data file.

Supplementary Table 1: Data collection and refinement statistics (molecular replacement)

	Dri1 (8GDW)	Dri1 + Heme (8GF4)	His21Ala Dri1 variant + Heme (8FM6)	His79Ala-Arg90Ala Dri1 variant + Heme (8GBK)
**Data collection**				
Space group	$P\ 2_1$	$P\ 6_1$	$P\ 6_5\ 2\ 2$	$P\ 4_3$
Cell dimensions				
a, *b*, *c* (Å)	51.72, 87.23, 57.24	42.98, 42.98, 379.48	74.66, 74.66, 211.19	91.82, 91.82, 144.31
α, β, γ (°)	90, 95, 90	90, 90, 120	90, 90, 120	90, 90, 90
**Anisotropic truncation by STARANISO**				
Diffraction limits from anisotropic analysis (Å)				
	--	--	0.894 a* - 0.447	a* = 2.802
	--	--	b* = 3.109	b* = 2.802
	--	--	c* = 2.755	c* = 2.685
Resolution (Å)	29.48 - 2.35 (2.4 - 2.35)*	50.0 – 3.0 (3.08 – 3.0) *	64.66 - 2.85 (3.05 - 2.85) *	29.94 - 2.9 (3.02 - 2.90) *
R_{merge}	0.055 (0.736)	0.092 (0.504)	0.148 (2.395)	0.135 (0.972)
R_{pim}			0.049 (0.691)	0.079 (0.570)
$I / \sigma I$	15.0 (1.9)	6.0 (1.3)	10.7 (1.2)	5.6 (1.6)
$CC_{1/2}$	0.999 (0.761)	1.0 (0.969)	0.999 (0.436)	0.994 (0.689)
Completeness (%)	99.0 (87.8)	92.6 (98.3)	93.6 (63.7)	93.5 (48.8)
Redundancy	5.3 (5.0)	3.5 (2.5)	10.0 (12.8)	3.9 (3.9)
Wilson B-factor (Å^2)	56.6	67.6	81.6	70.5
**Refinement**				
Resolution (Å)	2.35	3.0	2.85	2.9
No. reflections	20972 (1362)	7898 (830)	7169 (106)	26142 (2612)
R_{work} / R_{free}	0.197 / 0.247	0.201 / 0.279	0.217 / 0.269	0.311 / 0.326
No. atoms				
Protein	3096	2817	1480	5748
Ligand/ion	4	90	43	308
Water	34	3	0	0
B-factors				
Protein	66.7	70.8	76.74	74.02
Ligand/ion	55.3	67.0	105.81	74.59
Water	56.9	33.1	--	--

Supplementary Table 1 (continued)

Dri1 (8GDW)	Dri1 + Heme (8GF4)	His21Ala Dri1 variant + Heme (8FM6)	His79Ala-Arg90Ala Dri1 variant + Heme (8GBK)	Dri1 (8GDW)
**Ramachandran Plot**				
Ramachandran favored (%)	93.0	90.0	97.40	95.23
Ramachandran allowed (%)	6.0	9.0	2.60	4.77
Ramachandran outliers (%)	1	1	0.00	0.00
Rotamer outliers (%)	12	16	0.63	1.45
R.m.s. deviations				
Bond lengths (Å)	0.007	0.008	0.007	0.009
Bond angles (°)	1.5	2.0	0.94	1.24

*A single crystal was used for each structure.
*Values in parentheses are for highest-resolution shell.
*Additional parameters and statistics available in Methods.

Supplementary Table 2: Average radius of gyration (*Rg*) obtained from three independent SAXS-driven MD simulations using 3 different starting models (n=3 simulations).

Model	Average *Rg* (Å)	Standard Deviation
First frame from free-MD simulation of Dri1crystal structure	20.74	1.86
Last frame (100 ns) from free-MD simulation of Dri1crystal structure	21.54	1.79
First frame from free-MD simulation of *in silico* mutated Dri1 H21A	20.29	1.71
Last frame (100 ns) from free-MD simulation of *in silico* mutated Dri1 H21A	20.73	2.39
Dri1 H21A crystal structure	21.13	1.25
First frame from free-MD simulation of *in silico* mutated Dri1 H79A-R90A	20.81	1.69
Last frame (100 ns) from free-MD simulation of *in silico* mutated Dri1 H79A-R90A	20.13	2.46
Dri1 H79A-R90A crystal structure	19.98	1.89

Supplementary Table 3: SAS data acquisition, sample details, data analysis, modelling fitting and software used.

(*a*) Sample details			
	**Dri1**	**Dri1 H21A**	**Dri1 H79A-R90A**
Organism	Synechocystis sp. PCC 6803	Synechocystis sp. PCC 6803	Synechocystis sp. PCC 6803
Source (Catalogue No. or reference)			
Description: sequence (including Uniprot ID + uncleaved tags), bound ligands/modifications, *etc.*	P73129; MADPLTPAISDRICKHMNEDHASAIA LYAQVFGQQTDVTMAQMQAIDPTG MDLVVESEGGSKTIRIEFEQPLKDSE DAHQVLIAMAKQARSVGKNSAENL YFQ	P73129; MADPLTPAISDRICKHMNEDAASAIA LYAQVFGQQTDVTMAQMQAIDPTG MDLVVESEGGSKTIRIEFEQPLKDSE DAHQVLIAMAKQARSVGKNSAENL YFQ	P73129; MADPLTPAISDRICKHMNEDHASAIA LYAQVFGQQTDVTMAQMQAIDPTG MDLVVESEGGSKTIRIEFEQPLKDSE DAAQVLIAMAKQAASVGKNSAENL YFQ
Extinction coefficient ε (mM^{-1} cm^{-1})	2980 at 280 nm	2980 at 280 nm	2980 at 280 nm
Partial specific volume $\bar{\upsilon}$ (cm^3 g^{-1})	0.736	0.737	0.737
Molecular mass *M* from chemical composition (Da)	**- heme**: 11295.7<br>**+ heme**: 22591.4	**- heme**: 11229.7<br>**+ heme**: 22459.4	**- heme**: 11144.6<br>**+ heme**: 22289.2
For SEC-SAS, loading volume/concentration, (mg ml^{-1}) injection volume (µl), flow rate (ml min^{-1})	**Dri1**: 80 µl sample was injected at a 0.50 ml/min flow rate onto a GE Superdex 200 Increase 5/150 column<br>**Dri1 + heme**: 95 µl sample was injected at a 0.44 ml/min flow rate onto a GE Superdex 200 Increase 5/150 column<br>**apo-Dri1 + heme**: 90 µl sample was injected at a 0.50 ml/min flow rate onto a Biozen 3 µm dSEC-2, 200 Å column<br>**Zn-Dri1**: 90 µl sample was injected at a 0.50 ml/min flow rate onto a Biozen 3 µm dSEC-2, 200 Å column<br>**Zn-Dri1 + heme**: 90 µl sample was injected at a 0.50 ml/min flow rate onto a Biozen 3 µm dSEC-2, 200 Å column<br>**Co-Dri1 + heme**: 100 µl sample was injected at a 0.44 ml/min flow rate onto a GE Superdex 200 Increase 5/150 column	**- heme**: 95 µl sample was injected at a 0.44 ml/min flow rate onto a GE Superdex 200 Increase 5/150 column<br>**+ heme**: 85 µl sample was injected at a 0.35 ml/min flow rate onto a Biozen 3 µm dSEC-2, 200 Å column	**- heme**: 100 µl sample was injected at a 0.45 ml/min flow rate onto a GE Superdex 200 Increase 5/150 column<br>**+ heme**: 80 µl sample was injected at a 0.45 ml/min flow rate onto a GE Superdex 200 Increase 5/150 column
Solvent composition and source	50 mM Hepes, pH 7.5, 200 mM NaCl	50 mM Hepes, pH 7.5, 200 mM NaCl	50 mM Hepes, pH 7.5, 200 mM NaCl
(*b*) SAS data collection parameters			
Source, instrument and description or reference	NSLS-II; 16-ID (LiX)	NSLS-II; 16-ID (LiX)	NSLS-II; 16-ID (LiX)
Wavelength (Å)	**Dri1**: 0.08183 nm<br>**All other Dri1**: 0.08188 nm	**- heme**: 0.08188 nm<br>**+ heme**: 0.08172 nm	0.08199 nm
Beam geometry (size, sample-to-detector distance)	Beam size: 200 µm x 200 µm<br>**Dri1**: Pilatus3 X 1M and Pilatus3 X 900K duel detectors at sample-detector distances of 3.732 and 0.363 m<br>**Dri1 + heme**: sample-detector distances of 3.753 and 0.362 m<br>**apo-Dri1 + heme**: sample-detector distances of 3.760 and 0.343 m<br>**Zn-Dri1**: sample-detector distances of 3.760 and 0.343 m<br>**Zn-Dri1 + heme**: sample-detector distances of 3.760 and 0.343 m<br>**Co-Dri1 + heme**: sample-detector distances of 3.753 and 0.362 m	Beam size: 200 µm x 200 µm<br>**- heme**:<br>Pilatus3 X 1M and Pilatus3 X 900K duel detectors at sample-detector distances of 3.728 and 0.363 m<br>**+ heme**:<br>sample-detector distances of 3.727 and 0.343 m	Beam size: 200 µm x 200 µm<br>Pilatus3 X 1M and Pilatus3 X 900K duel detectors at sample-detector distances of 3.732 and 0.363 m
q-measurement range ($Å^{-1}$ or nm^{-1})	0.005 – 3.19 $Å^{-1}$	0.005 – 3.19 $Å^{-1}$	0.005 – 3.19 $Å^{-1}$
Method for monitoring radiation damage, X-ray dose where relevant	Yang, L., Antonelli, S., Chodankar, S., Byrnes, J., Lazo, E. & Qian, K. (2020). J. Synchrotron Rad. 27, 804-812.	Yang, L., Antonelli, S., Chodankar, S., Byrnes, J., Lazo, E. & Qian, K. (2020). J. Synchrotron Rad. 27, 804-812.	Yang, L., Antonelli, S., Chodankar, S., Byrnes, J., Lazo, E. & Qian, K. (2020). J. Synchrotron Rad. 27, 804-812.
Exposure time, number of exposures	**Dri1**: 7 x 2 second frames<br>**Dri1 + heme**: 12 x 2 second frames<br>apo-Dri1 + heme: 13 x 2 second frames<br>**Zn-Dri1**: 8 x 2 second frames<br>**Zn-Dri1 + heme**: 6 x 2 second frames<br>**Co-Dri1 + heme**: 13 x 2 second frames	**- heme**: 8 x 2 second frames<br>**+ heme**: 7 x 2 second frames	**- heme**: 9 x 2 second frames<br>**+ heme**: 11 x 2 second frames
Sample configuration including path length and flow rate where relevant	Yang, L., Antonelli, S., Chodankar, S., Byrnes, J., Lazo, E. & Qian, K. (2020). J. Synchrotron Rad. 27, 804-812.	Yang, L., Antonelli, S., Chodankar, S., Byrnes, J., Lazo, E. & Qian, K. (2020). J. Synchrotron Rad. 27, 804-812.	Yang, L., Antonelli, S., Chodankar, S., Byrnes, J., Lazo, E. & Qian, K. (2020). J. Synchrotron Rad. 27, 804-812.
Sample temperature (ºC)	4 °C	4 °C	4 °C

Supplementary Table 3 (Continued)

(*c*) Software employed for SAS data reduction, analysis, and interpretation	
SAS data reduction to sample–solvent scattering, and extrapo-Dri1-Dri1lation, merging, desmearing *etc.* as relevant	lixtools, py4xs
Calculation of ε from sequence	ExPASy ProtParam tool
Calculation of $\Delta\bar{\rho}$ and $\bar{\upsilon}$ values from chemical composition	NucProt Calculator (Database of Macromolecular Movements; www.molmovdb.org)
Basic analyses: Guinier, *P*(*r*), scattering particle volume (*e.g.* Porod volume V_P or volume of correlation V_c)	ATSAS; BioXTAS RAW
Shape/bead modelling	BioXTAS RAW; DENSS
Atomic structure modelling (homology, rigid body, ensemble)	SAXS-driven molecular dynamics
Modelling of missing sequence from PDB files	---
Molecular graphics	Pymol

(*d*) Structural parameters

Guinier Analysis	**Dri1**	**Dri1 H21A**	**Dri1 H79A-R90A**
I(0) (cm^{-1})	**Dri1**: 2.67 ± 0.01<br>**Dri1 + heme**: 2.41 ± 0.01<br>**apo-Dri1 + heme**: 9.33 ± 0.01<br>**Zn-Dri1**: 3.383 ± 0.009<br>**Zn-Dri1 + heme**: 14.93 ± 0.02<br>**Co-Dri1 + heme**: 6.17 ± 0.01	**- heme**: 5.05 ± 0.01<br>**+ heme**: 15.35 ± 0.02	**- heme**: 2.53 ± 0.01<br>**+ heme**: 3.88 ± 0.01
R_g (Å)	**Dri1**: 16.15 ± 0.12<br>**Dri1 + heme**: 19.40 ± 0.12<br>**apo-Dri1 + heme**: 19.86 ± 0.07<br>**Zn-Dri1**: 15.47 ± 0.14<br>**Zn-Dri1 + heme**: 19.67 ± 0.06<br>**Co-Dri1 + heme**: 19.66 ± 0.14	**- heme**: 15.82 ± 0.05<br>**+ heme**: 19.80 ± 0.05	**- heme**: 16.16 ± 0.09<br>**+ heme**: 19.66 ± 0.09
q-range ($Å^{-1}$)	**Dri1**: 0.0075 – 0.08<br>**Dri1 + heme**: 0.0075 – 0.065<br>**apo-Dri1 + heme**: 0.01 – 0.065<br>**Zn-Dri1**: 0.015 – 0.085<br>**Zn-Dri1 + heme**: 0.005 – 0.065<br>**Co-Dri1 + heme**: 0.005 – 0.065	**- heme**: 0.01 – 0.085<br>**+ heme**: 0.0075 - 0.065	**- heme**: 0.0125 – 0.08<br>**+ heme**: 0.015 – 0.065
Quality-of-fit parameter (R^2)	**Dri1**: 0.997<br>**Dri1 + heme**: 0.991<br>**apo-Dri1 + heme**: 0.999<br>**Zn-Dri1**: 0.992<br>**Zn-Dri1 + heme**: 0.992<br>**Co-Dri1 + heme**: 0.997	**- heme**: 0.995<br>**+ heme**: 0.996	**- heme**: 0.992<br>**+ heme**: 0.998
P(*r*) analysis	**Dri1**	**Dri1 H21A**	**Dri1 H79A-R90A**
I(0) (cm^{-1})	**Dri1**: 2.65 ± 0.01<br>**Dri1 + heme**: 2.41 ± 0.09<br>**apo-Dri1 + heme**: 9.28 ± 0.02<br>**Zn-Dri1**: 3.37 ± 0.01<br>**Zn-Dri1 + heme**: 14.88 ± 0.03<br>**Co-Dri1 + heme**: 6.16 ± 0.01	**- heme**: 5.05 ± 0.02<br>**+ heme**: 15.37 ± 0.03	**- heme**: 2.53 ± 0.01<br>**+ heme**: 3.86 ± 0.01
R_g (Å)	**Dri1**: 16.08 ± 0.1<br>**Dri1 + heme**: 19.53 ± 0.1<br>**apo-Dri1 + heme**: 19.76 ± 0.05<br>**Zn-Dri1**: 15.46 ± 0.07<br>**Zn-Dri1 + heme**: 19.65 ± 0.05<br>**Co-Dri1 + heme**: 19.64 ± 0.07	**- heme**: 16.05 ± 0.09<br>**+ heme**: 19.99 ± 0.05	**- heme**: 16.34 ± 0.11<br>**+ heme**: 19.6 ± 0.1
d_{max} (Å)	**Dri1**: 53<br>**Dri1 + heme**: 62<br>**apo-Dri1 + heme**: 63<br>**Zn-Dri1**: 50<br>**Zn-Dri1 + heme**: 62<br>**Co-Dri1 + heme**: 63	**- heme**: 57<br>**+ heme**: 65	**- heme**: 55<br>**+ heme**: 63
q-range ($Å^{-1}$)	**Dri1**: 0.005 – 1<br>**Dri1 + heme**: 0.0075 –1<br>**apo-Dri1 + heme**: 0.01 – 1<br>**Zn-Dri1**: 0.015 – 1<br>**Zn-Dri1 + heme**: 0.005 – 1<br>**Co-Dri1 + heme**: 0.005 – 1	**- heme**: 0.0150 – 1<br>**+ heme**: 0.0225 – 0.4	**- heme**: 0.015 – 1<br>**+ heme**: 0.0125 – 1
Quality-of-fit parameter (Total Estimate from 0 – 1, with 1 being ideal)	**Dri1**: 0.8118<br>**Dri1 + heme**: 0.8549<br>**apo-Dri1 + heme**: 0.8909<br>**Zn-Dri1**: 0.7991<br>**Zn-Dri1 + heme**: 0.9283<br>**Co-Dri1 + heme**: 0.8498	**- heme**: 0.8056<br>**+ heme**: 0.8708	**- heme**: 0.7988<br>**+ heme**: 0.8649
Volume (V_c; V_P)	**Dri1**: 149.9 $Å^2$; 12200 $Å^3$<br>**Dri1 + heme**: 211.0 $Å^2$; 22600 $Å^3$<br>**apo-Dri1 + heme**: 225.1 $Å^2$; 25600 $Å^3$<br>**Zn-Dri1**: 141.8 $Å^2$; 10400 $Å^3$<br>**Zn-Dri1 + heme**: 225.7 $Å^2$; 25700 $Å^3$<br>**Co-Dri1 + heme**: 224.4 $Å^2$; 25300 $Å^3$	**- heme**: 146.1 $Å^2$; 11600 $Å^3$<br>**+ heme**: 221.1$Å^2$; 25100 $Å^3$	**- heme**: 150.0 $Å^2$; 12600 $Å^3$<br>**+ heme**: 219.5 $Å^2$; 25500 $Å^3$

Supplementary Table 3 (Continued)

(*e*) Shape modelling results (a complete panel for each method)			
	**Dri1**	**Dri1 H21A**	**Dri1 H79A-R90A**
q-range for fitting	**Dri1**: 0.005 – 1<br>**Dri1 + heme**: 0.0075 –1<br>**apo-Dri1 + heme**: 0.01 – 1<br>**Zn-Dri1**: 0.015 – 1<br>**Zn-Dri1 + heme**: 0.005 – 1<br>**Co-Dri1 + heme**: 0.005 – 1	**- heme**: 0.015 – 1<br>**+ heme**: 0.0225 – 0.4	**- heme**: 0.015 – 1<br>**+ heme**: 0.0125 – 1
Symmetry/anisotropy assumptions	P1	P1	P1
Ambiguity score (AMBIMETER)	**Dri1**: 0.9031<br>**Dri1 + heme**: 1.362<br>**apo-Dri1 + heme**: 1.415<br>**Zn-Dri1**: 1.230<br>**Zn-Dri1 + heme**: 1.380<br>**Co-Dri1 + heme**: 1.380	**- heme**: 0.6990<br>**+ heme**: 1.580	**- heme**: 0.6021<br>**+ heme**: 1.079
χ^2 value/range	**Dri1**: 0.00145<br>**Dri1 + heme**: 0.08751<br>**apo-Dri1 + heme**: 0.277<br>**Zn-Dri1**: 0.02315<br>**Zn-Dri1 + heme**: 0.00093<br>**Co-Dri1 + heme**: 0.23731	**- heme**: 0.08388<br>**+ heme**: 0.03925	**- heme**: 0.07766<br>**+ heme**: 0.07331
Real-space correlation	**Dri1**: 0.51 ± 0.07<br>**Dri1 + heme**: 0.52 ± 0.04<br>**apo-Dri1 + heme**: 0.53 ± 0.07<br>**Zn-Dri1**: 0.47 ± 0.09<br>**Zn-Dri1 + heme**: 0.51 ± 0.07<br>**Co-Dri1 + heme**: 0.52 ± 0.06	**- heme**: 0.55 ± 0.06<br>**+ heme**: 0.64 ± 0.06	**- heme**: 0.54 ± 0.05<br>**+ heme**: 0.56 ± 0.06
Fourier Shell Correlation Resolution (Å)	**Dri1**: 24.7 ± 6.7<br>**Dri1 + heme**: 26.4 ± 5.2<br>**apo-Dri1 + heme**: 28.5 ± 6.7<br>**Zn-Dri1**: 25.5 ± 7.4<br>**Zn-Dri1 + heme**: 29.1 ± 5.7<br>**Co-Dri1 + heme**: 29.5 ± 6.5	**- heme**: 22.9 ± 4.8<br>**+ heme**: 27.5 ± 6.1	**- heme**: 25 ± 6<br>**+ heme**: 28.1 ± 6.3

(*f*) Atomistic modelling			
	**Dri1**	**Dri1 H21A**	**Dri1 H79A-R90A**
Method	SAXS-driven MD modelling	SAXS-driven MD modelling	SAXS-driven MD modelling
q-range for fitting	0.006-1.0	0.008-1.0	0.006-1.0
Symmetry assumptions	None	None	None
Any measures of model precision	None	None	None
χ^2 value/range	1.61	1.56	1.61
Relevant output parameters (*e.g.* predicted R_g/d_{max} values, weights for multi-state models, *etc.*)	19.85 Å	19.93 Å	19.34 Å

(*g*) Data and model deposition IDs			
	**Dri1**	**Dri1 H21A**	**Dri1 H79A-R90A**
	**Dri1**: SASDRH5<br>**Dri1 + heme**: SASDRJ5<br>**apo-Dri1 + heme**: SASDRG5<br>**Zn-Dri1**: SASDRK5<br>**Zn-Dri1 + heme**: SASDRL5<br>**Co-Dri1 + heme**: SASDRM5	**- heme**: SASDRF5<br>**+ heme**: SASDQS9	**- heme**: SASDRE5<br>**+ heme**: SASDRD5

Supplementary Table 4: Description of the molecular dynamics (MD) system setups.

Model	MD type	Simulation box dimensions (nm*nm*nm)	Number of atoms (Protein + heme)	Total number of water molecules	Salt concentration
Dri1	SAXS-driven MD	18.39035*18.39035*13.00393	3124	109744	200 mM NaCl
in silico mutated Dri1 H21A	SAXS-driven MD	18.39153 *18.39153 *13.00477	3110	109750	200 mM NaCl
in silico mutated Dri H79A-R90A	SAXS-driven MD	18.38393*18.38393*12.99939	3082	109756	200 mM NaCl
Dri1 H21A crystal structure	SAXS-driven MD	14.88431*14.88431*10.52480	3142	75538	200 mM NaCl
Dri1 H79A-R90A crystal structure	SAXS-driven MD	15.21765*15.21765*10.76050	3019	80767	200 mM NaCl
Dri1	Free-MD	18.38086*18.38086*12.99722	3124	109744	200 mM NaCl
in silico mutated Dri1 H16A	Free-MD	13.39901*13.39901*9.47453	3110	54871	200 mM NaCl
in silico mutated Dri1 H21A	Free-MD	18.37971*18.37971*12.99641	3110	109750	200 mM NaCl
in silico mutated Dri1 H79A	Free-MD	13.40379 *13.40379 *9.47791	3110	54868	200 mM NaCl
in silico mutated Dri1 H16A-H21A	Free-MD	13.40190*13.40190 *9.47657	3096	54871	200 mM NaCl
in silico mutated Dri1 H16A-H79A	Free-MD	9.34186*9.34186*6.60569	3096	17910	200 mM NaCl
in silico mutated Dri1 H21A-H79A	Free-MD	9.31698 *9.31698*6.58809	3096	17907	200 mM NaCl
in silico mutated Dri1 H79A-R90A	Free-MD	18.38817*18.38817*13.00239	3082	109756	200 mM NaCl

Supplementary Table 5: Molecular Dynamics simulations checklist

**Reliability and reproducibility checklist for molecular dynamics simulations**<br>***All boxes must be marked YES by acceptance unless an N/A option is available**	**Yes**	**N/A**	**Response**<br>**(Please state where this information can be found in the text)**
**1. Convergence of simulations and analysis**			
1a. Is an evaluation presented in the text to show that the property being measured has equilibrated in the simulations<br>(*e.g.* time-course analysis)?	☒		RMSD for free-MD and Radius of gyration for SAXS-driven MD over simulation time are provided in Supplementary Fig. 11.
1b. Then, is it described in the text how simulations are split into equilibration and production runs and how much data were analyzed from production runs?	☒		This information is available in the Methods section for molecular dynamics
1c. Are there at least 3 simulations per simulation condition with statistical analysis?	☒		Yes, three different simulations were performed for each SAXS-driven MD. The average Rg values obtained from 3 different simulations for each of the 3 starting models are provided in Supplementary Table 2.
1d. Is evidence provided in the text that the simulation results presented are independent of initial configuration?	☒		Yes, this is shown in Supplementary Fig. 4 and 9, where convergence was achieved using different starting models that were used for SAXS-driven MD.
**2. Connection to experiments**			
2a. Are calculations provided that can connect to experiments (*e.g.* loss or gain in function from mutagenesis, binding assays, NMR chemical shifts, J-couplings, SAXS curves, interaction distances or FRET distances, structure factors, diffusion coefficients, bulk modulus and other mechanical properties, *etc.*)?	☒		Yes, SAXS-driven MD simulations described in Methods section
**3. Method choice**			
3a. Is it described in the text what force field and water model are used and why?	☒		This information is available in the Methods section (Molecular dynamic simulations).
3b. Do simulations contain membranes, membrane proteins, intrinsically disordered proteins,	☐	☒	Response not needed if **N/A**

glycans, nucleic acids, polymers, or cryptic ligand binding?			
If 3b is **YES**, are enhanced sampling methods used?	☐	☒	Response not needed if **N/A**
If enhanced sampling methods are used, are the convergence criteria clearly stated?	☐		
If 3b is **YES**, is it explained in the text why or why not enhanced sampling methods are used?	☐		
**4. Code and reproducibility**			
4a. Is a table provided describing the system setup, such as simulation box dimensions, total number of atoms, total number of water molecules, salt concentration, lipid composition (number of molecules and type)?	☐		The table gathering this information has been added as Supplementary Table 3.
4b. Is it described in the text what simulation and analysis software and which versions are used?	☒		This information is available in the Methods section.
4c. Are initial coordinate and simulation input files and a coordinate file of the final output provided as supplementary files or in a public repository?	☐		This information has been uploaded on Zenodo (https://zenodo.org/records/10789761).
4d. Is there custom code or custom force field parameters?	☐	☒	Response not needed if **N/A**
If **YES**, are they provided as supplementary profiles or in a public repository?	☐		
