# Supplementary material for: A hemoprotein with a zinc-mirror heme site ties heme availability to carbon metabolism in cyanobacteria
Source: Nat Commun. 2024 Apr 12;15:3167. doi: 10.1038/s41467-024-47486-z (PMC11014987; doi:10.1038/s41467-024-47486-z)
Supplement: Supplementary file 3 — Description of Additional Supplementary Files [file 41467_2024_47486_MOESM3_ESM.pdf]

File name: Supplementary Data 1

Description: Protein list, alignment file used to build the phylogenetic tree, and Newick Tree associated with Figure 1a.

File name: Supplementary Data 2

Description: List of strains, plasmids, and primers used in this study.
